# Health students’ expectations of the ideal educational environment: a qualitative research

**Published:** 2014-10

**Authors:** TEAMUR AGHAMOLAEI, MANDANA SHIRAZI, IDEH DADGARAN, HOOMAN SHAHSAVARI, AMIN GHANBARNEJAD

**Affiliations:** 1 Department of Public Health, Health School, Hormozgan University of Medical Sciences, Bandar Abbas, Iran;; 2 Educational Development Center, Virtual school and Medical School, Tehran University of Medical Sciences, Tehran, Iran;; 3 Research in medical education, Education Development Center (EDC), Guilan University of Medical Sciences (GUMS), Rasht, Iran;; 4 Department of Medical Surgical Nursing, Nursing and Midwifery School, Tehran University of Medical Sciences, Tehran, Iran;; 5 Department of Public Health, Health School, Hormozgan University of Medical Sciences, Bandar Abbas, Iran

**Keywords:** Education, Environment, Students, Qualitative research

## Abstract

**Introduction**: Educational environment is an important determinant of students’ behavior and its elements are associated with academic achievement and course satisfaction. The aim of this study was to determine students’ expectations of the ideal educational environment.

**Methods**: This was a qualitative study with content analysis approach. Using a theoretical sampling method, we selected eight students from Health School of Hormozgan University of Medical Sciences, studying health education, public health, environmental health, occupational health and medical entomology. To collect data, semi-structured interviews were used and continued until reaching data saturation. Qualitative content analysis was used to analyze the data.

**Results: **Students' expectations of the ideal educational environment emerged in four main themes including school atmosphere, teaching, human aspects (with three subthemes including teachers, students, and school staff) and non-human aspects (with two subthemes including educational equipment and physical environment).

**Conclusion: **Educational environment is a multidimensional issue and to achieve an ideal educational environment, educational planners should meet the students' expectations of the school atmosphere, teaching, teachers, students, school staff, educational equipment and physical environment.

## Introduction

Nowadays there is a growing interest in the role of educational environments in medical education. Educational environment is one of the most important determinants of curriculum success and the quality of the learning environment markedly affects learning (1). Moreover, educational environment is an important determinant of students’ behavior and its elements are associated with academic achievement and course satisfaction (2).

Educational environment influences how, why and what students learn. It is crucial in success of the curriculum (3).To provide quality enhancement of the educational environment, higher educational institutions should create and implement a strategy for their higher school improvement (4).

Teaching has been recognized not only for providing information and the exchange of experiences, but also for creating the atmosphere and facilitating the learning environment. Students are expected to experience different learning activities in the school. The educational program is one of the most important determinants of the learning environment and the learning environment is one of the most important determinants of the behavior of all participants in the training (5).

In higher education, issues of a learning environment can be evaluated from different perspectives. Any school or educational institute has its own understanding of the learning environment. In order to implement a good and appropriate training, it is required to understand the concept of the educational environment to properly apply it in the school (6, 7).

One way to do this is to determine the students’ perceptions and their expectations of the educational environment. Students’ perceptions and expectations can be a good basis for reform and improvement of the quality of the educational environment. There is a direct relationship between the learning of students and their perceptions of the educational environment (8). Researches in the field of education showed that cognitive, motivational, affective and behavioral consequences of students are largely shaped by characteristics of the educational environment such as the learning atmosphere, educational quality and the structure of the classrooms (9, 10).

Since students’ perceptions and their expectations of the educational environment may be influenced by increasing the diversity of the student population, their different expectations and the specific conditions and facilities of the university (11), determining the students’ expectations of the educational environment and collecting basic information in order to improve the courses in any university is inevitable. In recent years the interest for assessing students' expectations and their perceptions of the learning environment in medical schools has increased. The studies regarding educational environment in Iran are more concerned with Medical schools, using mainly DREEM scale with a quantitative approach (12, 13). Moreover, no research has been conducted in Iran to study the students’ expectations of the ideal educational environment with a qualitative approach to describe hidden aspects of the educational environment. So the aim of this study was to determine students’ expectations of the ideal educational environment using a quantitative approach.

## Methods

**Research design:** This research was of a qualitative type, using a content analysis approach. Qualitative research can help researches to achieve a phenomenon experienced by participants. Qualitative content analysis can be used to determine the existence of certain words and concepts in the text or set of texts. The aim of qualitative content analysis is to classify information obtained from the manuscript of interviews, observations, and data obtained from video tapes.

**Background and Participants: **This study was conducted in Health School of Hormozgan University of Medical Sciences in Bandar Abbas, a city in the south of Iran. The target population included students studying health education, public health, environmental health, occupational health, and medical entomology at the Health School in the first semester of 2013. In the present research, eight students in different fields of study were selected through purposive sampling. The chief researcher selected the most informant and the appropriate participant as the first participant and ran the research (Table 1). Sampling went on until data saturation.

**Data collection**: To collect data, semi-structured interviews were used and continued until reaching data saturation. The face to face interviews were conducted in the health school. At the beginning of the interview, participants became familiar with the purpose of the study and their consent for voice recording was obtained. Each interview lasted about thirty minutes. Interview guides and field notes were used during the interviews.

According to the purpose of the study, interviews were started with questions such as “What are the characteristics of an ideal educational environment in your opinion?”, “What are the dimensions of an academic educational environment?”, “What are your expectations from an ideal educational environment” and continued according to the response of participants. During the interviews exploratory questions such as “give an example, can you explain more”, “what do you mean?”, “why do you think …?” and so on were used. Each interviewee was assigned a code from 1 to 8. As immediate registration of data is an important factor for the success of the researcher in the analysis of the data (14), interviews were typed word by word as soon as possible after the interview. For this purpose, at the first, the interviews were listened, typed word by word and matched again with the recorded data. It was performed in order to increase the accuracy of the recorded data and more control over the collected data.

**Data Analysis**: Qualitative content analysis with a conventional approach was used to analyze the data. In content analysis at the first, semantic units should be specified, and then the related codes should be extracted and categorized based on the similarity. Finally, in the case of having a high degree of abstraction, the themes can be determined. Content analysis method is used to verify the existence of certain words and concepts in the text for giving structure and discipline to the data (14). In a conventional approach, use of predetermined classes is avoided and classes and their names are allowed to directly come out of the data. To do so, the manuscripts of the interviews were read and the recorded data were listened to several times until an overall sense was attained. Then the manuscripts were read word by word and the codes were extracted. At the same time, the interviews were continued with other participants and coding of texts was continued and sub the codes were categorized within the general topics. Then the codes were classified in categories based on the similarity (15). The categories which were similar were classified in more general categories and each category was given a name.

**Validity and reliability of the data**: To ensure the validity and reliability of the data, the criteria of credibility, confirmability, and transferability were used (16). In this research the credibility obtained through continuous communication with the participants and prolonged engagement with the data. After primary coding, the manuscripts of the interviews were returned to the participants in order to ensure the agreement between researcher and them. Allocation of enough time to collect the data was another factor that was used to increase the reliability of the data. Students in all fields of study were included in the study. To verify the confirmability, the manuscripts of the interviews and the process of coding were peer reviewed and in most cases there was agreement among reviewers. To evaluate the transferability of the data, the collected data were shared with two students out of the research project who had similar positions as the target population (17) and in most cases the results were similar to their experiences.

**Ethical considerations**: The aim of the research and interview method was explained to the participants and informed consent for interview and recording the conversation was obtained.

**Table 1:** Demographic characteristic of the participants in the study

**Code**	**Age**	**Sex**	**Field of study**
1	31	Female	Health education
2	21	Female	Occupational health
3	20	Female	Public health
4	26	Male	Public health
5	21	Male	Occupational health
6	23	Female	Environmental health
7	21	Male	Environmental health
8	24	Male	Medical entomology

## Results

Based on the initial coding, 346 codes were obtained. These codes, based on the conceptual similarities and differences, were classified and summarized. Finally, the students' expectations from the ideal educational environment were classifies under four main themes including school atmosphere, teaching, human aspects (with three subthemes including teachers, students, and school staff) and non-human aspects (with two subthemes including educational equipment and physical environment) (Table 2).

**Table 2:** Main themes and subthemes of educational environment

**Row**	**Themes**	**Subthemes**
1	School atmosphere	
2	Teaching	
3	Human aspects	Teachers Students School staff
4	Non-human aspects	Educational equipment Physical environment

1. **School atmosphere**
The school atmosphere refers to the factors like providing appropriate conditions for learning, providing a stressless learning environment, presence of efficient regulatory system for monitoring the performance of the faculties and school security monitoring. One of the participants, emphasizing the necessity of existence of regulations for students and teachers said: "*The ideal educational environment should have enough regulations for both teachers and students; and not only for students*" (code 2).
Another participant described proper educational environment as a stressless educational environment. "*The proper educational environment is an environment which does not expose students to stress*" (code 5).
Another participant focused on learning as a criterion for judging the effectiveness of an educational environment and said: "*The first issue in the educational environment is learning and it is necessary to provide facilities to obtain it*" (code 8).
2. **Teaching**
  This theme reflects the factors related to the process of training and includes elements such as using modern methods of teaching like problem solving method and group discussion, using new and up to date resources in teaching, using the class time efficiently, teaching the subjects understandably, encouraging the students to actively participate in class, emphasizing the importance and practical application of taught subjects, and providing the opportunity for students to practice in the laboratory and field.
One of the students pointed to the use of the new and up to date scientific resources and appropriate teaching methods and said: “*New sources should be used in teaching and these sources should be taught through modern educational methods*” (code 1).
Another student pointed to the active atmosphere created by the teacher in the classroom and said: “*The efficiency of an active class is more than the efficiency of an inactive class*” (code 2).
3. **Human aspects**
  In this theme the emphasis is on the features and tasks of teachers, students, and staff in an educational environment. This theme was classified into three subthemes including teachers, students, and staff.
  - **Teachers**
    This subtheme reflects characteristics and roles of teachers in the learning environment. This subtheme includes elements such as availability of experienced and skilled teachers in the school, creation of a comfortable atmosphere in the classrooms, ability of teachers to manage the classes, respectful behavior with students, motivation development for the students to study and learn, ability of teachers to communicate with the students, preparedness of teachers to teach the subjects, convenient access to teachers and the use of appropriate methods to evaluate the students.
One of the participants described the characteristics of teachers in an ideal educational environment and said: “*A teacher should be able to motivate the students, have good speaking skills, teach the subjects understandably and motivate the students to participate in the class*” (code 3). This participant also emphasized the role of the teachers in creating a positive atmosphere in the class and said: “*The conditions that a teacher creates in the classroom should not be rigid and the students should not feel uncomfortable. The class environment should be comfortable so that students could easily ask their questions*” (code 3).
Another participant emphasized the optimal use of class time and said: “*The teacher should be present at the class on time, leave the class on time and use the class* *time effeciently*” (code 5).
One of the participants, regarding the characteristics of the teachers, said: “*The teachers should be knowledgeable and have good speaking skills. Some of the teachers have enough knowledge but they can’t transfer it to the students*” (code 6).
Another participant emphasized the communication skills of the teachers and said: “*Teachers* *must be able* *to communicate* *with the students properly, allocate* *enough time* *to the students and response their questions, and assign research projects to the students*” (code 7). Another participant in this regard said: *“The effectiveness of communication between the teacher and student is one of the most important factors which affect the learning. If a teacher could establish and have a good and intimate relationship with the students, the students’ performance will increase a lot”* (code 8).
  - **Students**
    This subtheme reflects characteristics and roles of the students in the learning environment. This subtheme includes elements such as to attend timely and regularly in the classes, to be interested in the field of study, to behave respectfully in the school, to participate in the course subjects actively, to have academic ability in the field of study, to be familiar with the scientific databases, to have sense of cooperation, to have enough motivation for learning and to use educational facilities efficiently.
Emphasizing the collaborative work, one of the participants said: “*Students should have the sense of teamwork. Unfortunately, I have seen that teamwork assignments are often done by only one or two students of the team”* (code 3).
Another participant with an emphasis on the active role of the students in acquiring knowledge said: “*Acquiring knowledge is not a passive process; the students should search the scientific resources, try hard and learn actively*” (code 5).
  - **School Staff**
    This subtheme reflects the characteristics and roles of the staff in the learning environment. This subtheme includes elements such as having commitment and responsibility for doing one’s job, being experienced and knowledgeable, having good communication skills, behaving respectfully with the students and being patient and tolerant.
One of the participants, regarding the expectations from the staff in an efficient learning environment, said: “*The staff should do their jobs well, communicate and behave well with the students, guide the students and help them to solve their problems*” (code 4).
4. **Non-human aspects**
  In this theme the emphasis is on the conditions and characteristics of the equipment and facilities, and the physical environment of the learning environment. This theme was classified into two subthemes including educational equipment and physical environment.
  - **Educational equipment**
    This subtheme reflects the educational equipment and facilities in the school and includes elements such as appropriate educational aids and comfortable seats in the classrooms, presence of up to date scientific sources in the library, convenient access to scientific sources and databases, having high internet speed, and adequate equipment and facilities in the library, computer site, and laboratories.
One of participants said: “*The scientific sources in the library should be up to date; Laboratory facilities should be adequate for students and provide opportunities for practical work*” (code 3).
Another participant, regarding the Internet, said: “*Internet speed should be high, adequate number of computers should be in the computer site and the students should have access to the Internet only for doing scientific and research work*” (code 7).
Another participant emphasized the suitability of the classroom chairs and said: “*Ergonomic characteristics of the chairs* *in the classrooms* *should* *be* *good and during class times students should not feel uncomfortable*” (code 8).
  - **Physical environment**
    This subtheme reflects characteristics and conditions of physical environment of the educational institute. This subtheme includes elements such as quietness of classrooms, the suitability of the physical environment of the classrooms, suitable arrangement of classrooms seats, quietness of the library, quite physical space in the library, computer site and laboratories for students and aesthetic aspects of the school yard.
One of the participant emphasized the role of school building in shaping the attitudes of students toward the learning environment and stated: “*The first time that a student enters the university, he/she sees the campus. He doesn't know anything about the teachers and school facilities and his/her early attitudes toward the learning environment is shaped by this first visit of the campus*” (code 5).
Another participant, regarding the physical environment of the library and classrooms, said: “*The library and classrooms should be quiet with enough physical space for students. A classroom should be neither large nor small*” (code 8).

## Discussion

The aim of this study was to determine the students’ expectations of the ideal educational environment in the Health School of Hormozgan University of Medical Sciences. The students' expectations of the ideal educational environment were categorized in four main themes including school atmosphere, teaching, human aspects (with three subthemes including teachers, students, and school staff) and non-human aspects (with two subthemes including educational equipments and physical environment).

The ideal school atmosphere, from students’ viewpoints, was the one which provides appropriate conditions for learning, provides a stressless learning environment, and has an efficient regulatory system for monitoring the performance of the faculties. In general, school atmosphere reflects the viewpoints of the students regarding the surrounding atmosphere in the learning environment. It also reflects the effectiveness of the educational programs (18). One of the students’ expectations regarding the educational environment was creating a stressless environment. An indirect relationship between stress and academic performance of students has been shown (19). Students who report higher stress levels perceive lack of self-confidence and poor process control in their learning (20). The educational atmosphere is a determining factor which motivates students for learning (21). Considering the students’ expectations of the school atmosphere can improve the educational environment.

The theme of teaching reflects the factors related to the process of training. Learning is a complex process and is affected mostly by the educational environment and the context of the school (22). Some of the students’ expectations that should be emphasized to achieve efficient learning include use of modern methods of teaching, use of new and up to date scientific sources and emphasis on the importance and practical applications of the taught subjects. The results of this study showed that students had high levels of expectations of the educational environment and their faculty. Previous studies support this finding (11, 23, 24). It is important for the students that their teachers be competent and prepared well for teaching. So in organizing approaches and planning methods of teaching and learning, these expectations are recommended to be considered.

The subtheme of teachers reflects characteristics and roles of the teachers in the learning environment. The main task of the university is to train and the teachers are one of the key and essential components of the training process (25). The teacher plays an important role in the realization of educational goals and can compensate for the shortage of the scientific sources. The performance and features of the teacher can influence the learning process and educational goal achievement. Facilitating the learning process, guiding the students, training students on the moral issues, and improving the interpersonal communication, and crisis management are some of the teachers’ roles in the educational environment (26). Because of rapid changes in medical education, faculties are necessary to be up to date and have enough skill and ability to handle their duties. Moreover, they should use the time efficiently and use modern and active teaching methods such as teaching in small groups, problem solving, group discussion and computer based methods (27).

The subtheme of students reflects characteristics and role of the students in the learning environment. This subtheme represents the views of students about their academic abilities and skills as well as the duties and activities that are expected from the students in a learning environment. One of the most important factors which affects the academic success of the students is their study and learning skills (28). Students’ success and academic achievement requires having a regular study program, using reliable scientific sources, having constant effort and using appropriate methods to study and learn academic subjects. In this subtheme, familiarizing the students with the scientific databases, creating a sense of cooperation among the students and emphasizing efficient use of educational facilities should be considered primarily.

The subtheme of school staff reflects characteristics and roles of the staff in the learning environment. To have commitment and responsibility for doing the tasks, to be experienced and knowledgeable, to have good communication skills, to behave respectfully with students and to be patient and tolerant are the most expectations the students had from the staff in the educational environment. 

In the theme of non-human aspects the emphasis is on the conditions and characteristics of the equipment and facilities, and the physical environment of the educational environment. In our study, this theme was classified into two subthemes including educational equipments and physical environment. The subtheme of educational equipments reflects the educational equipments and facilities in the school. Educational aids were under this subtheme. Teachers should have a plan and be trained for using them (29). Also, when using the educational aids, teachers should consider their effectiveness (30). In this subtheme preparing up to date scientific sources in the library with convenient access to them, providing appropriate equipment in the classrooms, library and computer site, having access to high speed Internet, and having convenient access to scientific databases should be considered primarily.

The subtheme of physical environment reflects characteristics and conditions of physical environment of the educational institute. The learning environment is not limited to interaction between the instructor and the students, and teaching and learning activities, but it also includes good physical structures and facilities provided by the university (31). Physical environment as one of the components of the educational environment has an important effect on the quality of education and it is one of the most important factors in the learning process (32, 33). Regarding the physical environment some factors such as temperature, lighting, ventilation and the arrangement of seats should be considered (33). In this subtheme, providing a quiet and large enough space in the library, computer site and laboratories and improving the classrooms’ temperature, light and color should be considered primarily.

## Conclusion

The results of this study showed that educational environment is a multidimensional issue and consists of anything that appears to be in the classroom, department and the university. Moreover, the level of the expectations of the students from the educational environment is very high. To achieve an ideal educational environment, educational planners should meet the students' expectations of the school atmosphere, teaching, teachers, students, school staff, educational equipments and physical environment. 

## References

[1] Makhdoom N (2009). Assessment of the quality of educational climate during undergraduate clinical teaching years in the college of medicine, Taibah University. Journal of Taibah University of Medical Sciences.

[2] Soemantri D, Herrera C, Riquelme A (2010). Measuring the educational environment in health professions studies: A systematic review. Med Teach.

[3] Al-Rukban MO, Khalil MS, Al-Zalabani A (2010). Learning environment in medical schools adopting different educational strategies. Educ Res Rev.

[4] Stukalina Y (2012). Addressing service quality issues in higher education: the educational environment evaluation from the students' perspective. Technological and economic development of economy.

[5] Demirören M, Palaoglu Ö, Kemahli S, Özyurda F, Ayhan I (2008). Perceptions of students in different phases of medical education of educational environment: Ankara University Faculty of Medicine. Med Educ Online.

[6] Lizzio A, Wilson K, Simons R (2002). University students' perceptions of the learning environment and academic outcomes: implications for theory and practice. Studies in Higher education.

[7] Roff S (2005). The Dundee Ready Educational Environment Measure (DREEM)-a generic instrument for measuring students' perceptions of undergraduate health professions curricula. Med Teach.

[8] Mayya S, Roff S (2004). Students' perceptions of educational environment: a comparison of academic achievers and under-achievers at Kasturba Medical College, India. Educ Health (Abingdon).

[9] Lüdtke O, Robitzsch A, Trautwein U, Kunter M (2009). Assessing the impact of learning environments: How to use student ratings of classroom or school characteristics in multilevel modeling. Contemporary Educational Psychology.

[10] Karabenick SA (2004). Perceived Achievement Goal Structure and College Student Help Seeking. Journal of Educational Psychology.

[11] Miles S, Leinster SJ (2007). Medical students' perceptions of their educational environment: expected versus actual perceptions. Med Educ.

[12] Aghamolaei T, Fazel I (2010). Medical students' perceptions of the educational environment at an Iranian Medical Sciences University. BMC Medical Education.

[13] Hamid B, Faroukh A, Mohammadhosein B (2013). Nursing Students' Perceptions of their Educational Environment Based on DREEM Model in an Iranian University. Malays J Med Sci.

[14] Adib Hajbagheri M, Parvizi R, Salsaly M (2007). Qualitative Research Methods.

[15] Hsieh HF, Shannon SE (2005). Three approaches to qualitative content analysis. Qualitative health research.

[16] Polit DF, Beck CT (2004). Nursing research: principles and practice.

[17] Speziale HS, Streubert HJS, Carpenter DR (2011). Qualitative research in nursing: Advancing the humanistic imperative.

[18] Genn J (2001). AMEE Medical Education Guide No. 23 (Part 1): Curriculum, environment, climate, quality and change in medical education—a unifying perspective. Med Teach.

[19] Roff S, McAleer S (2001). What is educational climate?. Med Teach.

[20] Dalband M, Farhadinasab A (2007). Evaluation of stress-inducing factors of educational environment in Hamadan Dentistry School's students. Scientific journal of Hamadan University of Medical Sciences and Health Services.

[21] Varma R, Tiyagi E, Gupta JK (2005). Determining the quality of educational climate across multiple undergraduate teaching sites using the DREEM inventory. BMC Med Educ.

[22] Cleary TS (2001). Indicators of Quality. Planning for Higher Education.

[23] Amin Z, Tani M, Hoon Eng K, Samarasekara DD, Huak CY (2009). Motivation, study habits, and expectations of medical students in Singapore. Med Teach.

[24] Draper C, Louw G (2007). What is medicine and what is a doctor? Medical students' perceptions and expectations of their academic and professional career. Med Teach.

[25] Bland CJ, Wersal L, VanLoy W, Jacott W (2002). Evaluating faculty performance: a systematically designed and assessed approach. Acad Med.

[26] Benor DE (2000). Faculty development, teacher training and teacher accreditation in medical education: twenty years from now. Med Teach.

[27] Wilkerson L, Irby DM (1998). Strategies for improving teaching practices: a comprehensive approach to faculty development. Acad Med.

[28] Murray B (1998). Getting smart about learning is her lesson. APA Monitor.

[29] Rasul S, Bukhsh Q, Batool S (2011). A study to analyze the effectiveness of audio visual aids in teaching learning process at uvniversity level. Procedia-Social and Behavioral Sciences.

[30] Yin C, Li C, Ai L (2010). The analysis of educational equipment cost and efficiency. Shiyan Jishu yu Guanli.

[31] Harden R (2001). The learning environment and the curriculum. Med Teach.

[32] Hutchinson L (2003). ABC of learning and teaching: Educational environment. BMJ.

[33] Mackway-Jones K, Walker M, Council R, Britain G (1999). The pocket guide to teaching for medical instructors.

